# Distribution and biological role of the oligopeptide-binding protein (OppA) in *Xanthomonas* species

**DOI:** 10.1590/S1415-47572010005000049

**Published:** 2010-06-01

**Authors:** Elisa E. Oshiro, Milene B. Tavares, Celso F. Suzuki, Daniel C. Pimenta, Claudia B. Angeli, Julio C. F. de Oliveira, Maria I. T. Ferro, Luis C. S. Ferreira, Rita C. C. Ferreira

**Affiliations:** 1 Departamento de Microbiologia, Universidade de São Paulo, São Paulo, SPBrazil; 2 Laboratório de Biofísica e Bioquímica, Instituto Butantan, São Paulo, SPBrazil; 3 Departamento de Parasitologia, Universidade de São Paulo, São Paulo, SPBrazil; 4 Universidade Federal de São Paulo, Diadema, SPBrazil; 5 Faculdade de Veterinária e Ciências Agrárias, Universidade Estadual Paulista Júlio de Mesquita Filho, Jaboticabal, SPBrazil

**Keywords:** *Xanthomonas*, *Xac*, OppA, oligopeptide uptake system, ABC transporter

## Abstract

In this study we investigated the prevalence of the *oppA* gene, encoding the oligopeptide binding protein (OppA) of the major bacterial oligopeptide uptake system (Opp), in different species of the genus *Xanthomonas*. The *oppA* gene was detected in two *Xanthomonas axonopodis* strains among eight tested *Xanthomonas* species. The generation of an isogenic *oppA*-knockout derivative of the *Xac* 306 strain, showed that the OppA protein neither plays a relevant role in oligopeptide uptake nor contributes to the infectivity and multiplication of the bacterial strain in leaves of sweet orange (*Citrus sinensis*) and Rangpur lime (*Citrus limonia*). Taken together these results suggest that the *oppA* gene has a recent evolutionary history in the genus and does not contribute in the physiology or pathogenesis of *X. axonopodis*.

## Introduction

Oligopeptides play important roles in bacterial nutrition, representing important sources of nitrogen, carbon and other elements. They are also involved in intercellular gene signaling processes, such as those involved in chemotaxis, conjugation, spore formation and the development of the competence state (Detmers *et al.*, 2001). Three distinct oligopeptide uptake systems, specifically committed to the transport of dipeptides (Dpp), tripeptides (Tpp) and oligopeptides (Opp), have been characterized in *Escherichia coli,Salmonella enterica* pv Typhimurum and *Sinorhizobium meliloti* (Hiles *et al.*, 1987; Nogales *et al.*, 2009).

In gram-negative bacteria, the Opp system belonging to the ATP-binding cassette (ABC) transporter family, comprises various functional and structural domains, *viz.*, the substrate binding protein in the periplasm (OppA), two transmembrane pore-forming proteins (OppB and OppC), and the two membrane-associated ATPases (OppD and OppF) which generate energy from ATP hydrolysis required for the transport process. The *opp* genes are frequently organized as a polycistronic operon (*oppABCD/F*), in which the binding component (OppA) is usually expressed at a higher stoichiometric ratio compared to the other components (Higgins and Hardie, 1983; Hiles *et al.*, 1987; Monnet, 2003). Besides a role in nutrition, the Opp system participates in various physiological processes, such as the recycling of cell-wall peptides, quorum sensing and adhesion to host cells, genetic competence and sporulation (Rudner *et al.*, 1991; Cundell *et al.*, 1995; Alloing *et al.*, 1998; Claverys *et al.*, 2000; Detmers *et al.*, 2001). Opp-encoding genes are found in approximately 50% of those bacterial species with available genomic sequences, although as yet, in only a few cases have their specific roles, either in physiology or pathogenesis, been investigated.

The genus *Xanthomonas* is comprised of a ubiquitous group of bacterial pathogens which infect crops of major economical interest, such as orange, grape, rice, soy bean and bean, among others (Chan and Goodwin, 1999). Genome sequencing projects of *Xanthomonas* species have highlighted the multiplicity of ABC transporters, particularly those responsible for nutrient uptake (Silva *et al.*, 2002; Qian *et al.*, 2005; Lee *et al.*, 2005; Thieme *et al.*, 2005; Salzberg *et al.*, 2008; Vorhölter *et al.*, 2008). So far, *opp* genes have been detected in the *X. axonopodis* pv. citri 306 (Vauterin *et al.*, 1995) (*Xac*) strain, but not in three *X. campestris* pv. campestris (*Xcc*) strains (Silva *et al.*, 2002; Qian *et al.*, 2005; Vorhölter *et al.*, 2008), neither in the genomes of *X. oryzae* pv. oryzae *(Xoo)* (Lee *et al.*, 2005; Salzberg *et al.*, 2008) and *X. campestris* pv. vesicatoria *(Xcv)* (Thieme *et al.*, 2005). These observations indicate that the Opp system may have a specific physiological role in *Xac* strains and may affect features like pathogenicity and host selectivity.

In the present study, we investigated the distribution of the *oppA* gene among different *Xanthomonas* species. In addition we evaluated the putative physiological role of OppA, namely that of the oligopeptide-binding component, in the *Xac* 306 strain, based on the generation of a specific knockout mutant. The present evidence demonstrated that the *oppA* gene is restricted to two *X. axonopodis* strains and does not play a specific role in the virulence of this phytopathogen.

## Material and Methods

###  Bacterial strains and growth conditions

All the bacterial strains used in this work are listed in Table 1. Bacterial strains were routinely cultivated in Circle Grow (CG) (Bio 101) broth at 30 °C. For the oligopeptide uptake assays, the strains were cultivated overnight in M9 minimal medium supplemented with proline, methionine, histidine, tryptophan (100 μg mL^-1^ each), or in the same medium with proline replaced by the YPLG peptide (0.5 μg mL^-1^).

###  Construction of a *Xac oppA*-knockout strain

The generation of an *oppA*-knockout *Xac* 306 derivative strain, denominated *XoppA2*, has already been described (Oshiro *et al.*, 2006).

###  Gene screening and expression studies

Genomic DNA from all the *Xanthomonas* isolates was isolated, according to the procedure described by Llop *et al.* (1999). The *oppA* gene was amplified with genomic DNA, as template and the oligonucleotides Fw 5'- CGGC GCTCGGGTACCGTGGCGTTGGCGGTGCTG-3' and Rv 5'- GGCGGATCTA GATCAGTGGTGGTGGTGG TGTTTGCTCACCCAGGCGTC- 3', based on the reported *opp* operon sequence (Silva *et al.*, 2002). For Southern-blot analysis, genomic DNA from the tested samples was digested with *Pst* I, and then transferred to a nylon membrane (Hybond-N, Amersham Biosciences). A probe for the *oppA* gene was synthesized with [α^32^P]-dCTP, by random primer labeling, using the PCR generated DNA fragment described above. The labeled probe was hybridized with the membrane at 42 °C for 16-20 h before exposure to autoradiography films (Kodak T-MAT G/RA film). Western-blot analyses were carried out with whole cell proteins sorted in 12.5% (w/v) acrylamide gels, followed by transference to nitrocellulose membranes (Millipore) and development of bands reacting with polyclonal monospecific anti-OppA antibody, as previously described (Moutran *et al.*, 2004). The anti-OppA serum was raised in mice parenterally immunized with a His-tagged protein produced in *E. coli* transformed with a recombinant pET-28a vector carrying the *Xac oppA* gene without the native signal peptide (Moutran *et al.*, 2004).

###  Oligopeptide uptake assay

*Xac* 306 and *XoppA2* cells, grown overnight, were harvested by centrifugation, washed twice with saline and inoculated (1:100) in the M9 medium without proline and with the tetrapeptide YPLG (Sigma) (0.5 μg mL^-1^). After various incubation periods at 28 °C, aliquots of the culture supernatant were harvested and dried by vacuum centrifugation. The peptides were extracted with cold methanol and submitted to RP-HPLC separation (LC 10A-VP binary Shimadzu HPLC system). Each fraction was analyzed in an ESI mass spectrometer (LCQDuo, ThermoFinnigan, USA), equipped with a nanospray source and connected to a nanoHPLC system (UltiMate HPLC System, LC Packings, Dionex, USA). The samples were introduced into the spectrometer by a flow rate at 1 μL/min, and then diluted in a solution of 5% acetonitrile and 0.2% formic acid. The spray voltage was kept at 1.8 kV, capillary voltage at 46 V, capillary temperature at 180 °C and tube lens offset at -5 V. MS spectra were collected in centroid mode in the 50 to 2000 *m*/*z* range.

###  Protease assays

Cell-free culture medium aliquots of *Xac* 306 and *XoppA2* strains prepared in M9 medium, were incubated with YPLG (0.5 μg mL^-1^) for different periods at 20-28 °C. The presence of the tetrapeptide was monitored by RP-HPLC fractionation, and the samples analyzed by mass spectrometry, as described above. The same experiments were repeated in the presence of EDTA (ethylenediaminetetraacetic acid) added to the medium aliquots at a final concentration of 1 mM.

###  Plant infection experiments

Cells of the *Xac* 306 and *XoppA2* strains were diluted in sterile destilled water to a final absorbance (OD_600_) of 0.3. Aliquots (100 μL) of cell suspensions were inoculated into the leaves of two sensitive citrus hosts, namely sweet orange (*Citrus sinensis*) and Rangpur lime (*Citrus limonia*), through injuries in the leaf surface. Infiltrations were carried out at the lower part of the leaf using a needleless syringe, as previously reported by Laia *et al.* (2009).

###  Studies of *in vivo* growth kinetics

*In vivo* growth tests of *Xac* strains were carried out with sweet orange (*C. sinensis*). The whole procedure followed the experimental conditions described by Laia *et al.* (2009). Five discs of at least 6 different leaves were assayed for each time point and for each tested bacterial strain.

## Results

**Figure 1:**
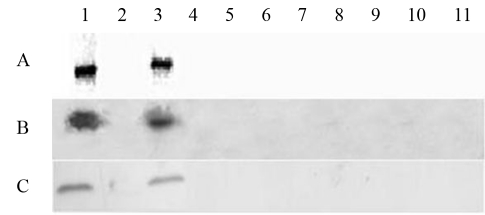
Detection of *oppA* gene and encoded protein (OppA) among different *Xanthomonas* species or strains. (A) The *oppA* gene was detected by PCR amplification with primers annealing with the genome sequence reported for the *Xac* 306 strain. Samples: 1, *Xac* 306 strain; 2, *Xaa* 409 strain; 3, *Xaa* 381 strain; 4, *Xcc* ATCC33913; 5, *X. bromi* LMG947; 6, *X. codiaei* LMG8678; 7, *X. sacchari* LMG739; 8, *Xcv* LMG911; 9, *X. pisi* IBSBF1356; 10, *X. theicolai* LMG8684; 11, *X. melonis* LMG8670*.* (B) Detection of the *oppA* gene by Southern blotting with a probe generated with the *oppA* gene of the *Xac* 306 strain. Sample order according to the distribution in panel (A). (C) Detection of the OppA protein in immuno blots, carried out with anti-OppA serum and periplasmic proteins of *Xanthomonas* species and strains. Sample order according to the distribution in panel (A).

*Opp* genes have only been discovered in the genome of the *Xac* 306 strain (Silva *et al.*, 2002). Moreover, no orthologs have been detected in genomes of the *Xcc* ATCC33913, 8004 and B100 strains (Silva *et al.*, 2002; Qian *et al.*, 2005; Vorhölter *et al.*, 2008), the *Xcv* 85-10 strain (Thieme *et al.*, 2005), nor the *Xoo* strains KACC10331 (Lee *et al.*, 2005), PX099^A^ (Salzberg *et al.*, 2008) and MAF311018 (GenBank accession number AP008229). These results indicate that *opp* genes are restricted to *Xac* and may have a specific physiological role, such as host specificity, in this bacterial species. To further investigate the distribution of the Opp system in *Xanthomonas* species, the *oppA* gene was screened in eight additional *Xanthomonas* species without known genome sequences, including one additional *Xcv* strain, six isolates of different *Xanthomonas* species (*X. bromi, X. codiaei,X. sacchari, X. pisi,X. theicola,* and *X. melonis,* and two *X. axonopodis pv aurantifolii (Xaa)* strains (409 and 381). As shown in Figure 1, the *oppA* gene was detected only in the *Xac* 306 and *Xaa* 381 strains, previously classified within the citrus pathovar, but currently, and according to the low DNA homology with *Xac* strains, considered as belonging to a different pathovar altogether (Schaad *et al.*, 2005). The *oppA* gene detected in both strains were actively transcribed and translated during *in vitro* growth. This was demonstrated by Western-blot analysis carried out with a specific anti-OppA polyclonal antibody generated in mice immunized with purified recombinant OppA protein from the *Xac* 306 strain. As shown in Figure 1C, the anti-OppA serum recognized a reactive protein band in the periplasm fraction of the two *X. axonopodis* strains (*Xac* 306 and *Xaa* 381), but not in extracts of any other *Xanthomonas* species or strain. The expression of OppA protein was apparently constitutive, since no significant difference in its expression was detected in whole-cell extracts of cells kept under different culture medium conditions, supplemented or not with oligopeptides or leaf extracts of susceptible citrus hosts (data not shown).

The restricted distribution of the *oppA* gene in the *Xanthomonas* genus suggests that the Opp system plays a specific physiological role in these two strains, but not in the other tested species and strains. In order to investigate the putative role of OppA in the uptake of oligopeptides by *X. axonopodis* strains, we generated an isogenic *oppA* defective knockout mutant strain (*XoppA2*) by a gene replacement approach in which the native gene was replaced by non-functional knockout alleles.

**Figure 2:**
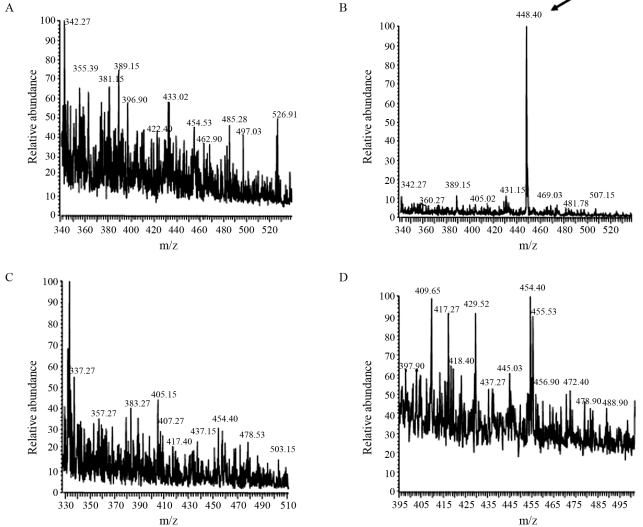
Oligopeptide uptake by *E. coli* K12 and *Xac* 306 strains. The profiles show the relative abundance versus mass spectrum (m/z = 448.4) corresponding to YLPG. (A) Profile detected in culture supernatant of *E. coli* SS320 strain in which no signal can be detected at m/z = 448.4 (range 2 x 10^3^). (B) Peptide profile detected in culture supernants of *E. coli* SS5013 (*opp* defective) strain, in which the presence of the tetrapeptide is clearly visible, as indicated by the arrow (range 2 x 10^5^). (C) Culture supernatant of the *Xac* 306 strain, in which no matching ion corresponding to YLPG could be detected (range 5 x 10^3^). (D) Culture supernatant of the *XoppA2* strain, in which no matching ion corresponding to YLPG could be detected (range 8 x 10^2^). The arrow points to the ion corresponding to the YLPG peptide.

The uptake of a synthetic tetrapeptide (YPLG) was monitored by mass spectrometry, using both the parental *Xac* 306 strain and the OppA-deficient *XoppA2* strain based on the removal of the synthetic peptide from the culture medium during bacterial cell growth. The choice of the substrate peptide was based mainly on molecular modeling studies and docking analyses of different peptides to the *Xac* OppA structural model (Moutran *et al.*, 2007). In order to validate the experimental approach, we employed two *E. coli* strains: one (the SS320 strain) proficient in the *opp* operon, and the other (the SS5013 strain) being an isogenic derivative deleted in the entire *opp* operon (Andrews and Short, 1985). As indicated in Figures 2A and 2B, the oligopeptides were efficiently removed from the culture supernatant by the *E.coli* SS320 strain, but not by the *opp*-deficient SS5013 strain. On the other hand, when the same experiment was repeated with *Xac* 306 and its isogenic *oppA*^*-*^ derivative, *XoppA2,* no tetrapeptide was detected in the culture supernatants of either strain, after incubation periods ranging from 12 to 24 h at 28 °C (Figure 2C,D). This might be explained, either by the presence of an alternative Opp-independent oligopeptide uptake system in the *Xac* 306 strain, or by the production and secretion of proteases in the culture media.

**Figure 3:**
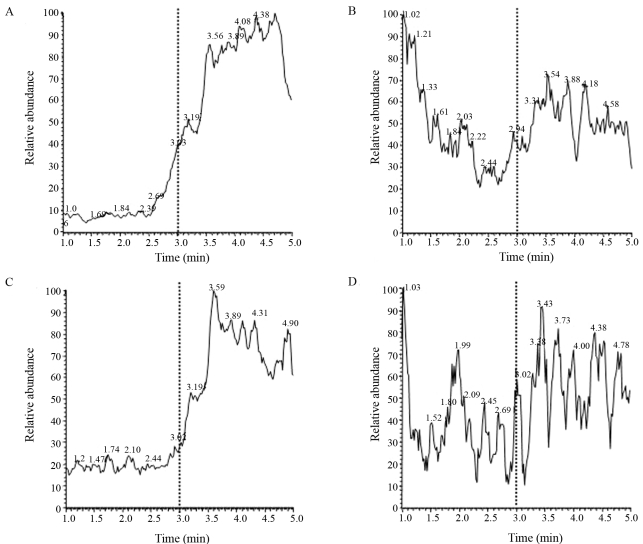
Proteolytic degradation of the tetrapeptide in culture supernatants of *Xac* 306 and *XoppA2* strains. The profiles show relative abundance versus time, with the YLPG peptide elutin after 3 min. Profiles detected in culture supernatant of the *Xac* 306 strain (A) with EDTA (range of 2.6 x 10^4^) or (B) without EDTA (range of 1.2 x 10^5^). Profiles detected in culture supernatant of the *XoppA2* strain (C) with EDTA (range of 7.6 x 10^3^) or (D) without EDTA (range of 1.5 x 10^5^). In both samples treated with EDTA, the absorbance peak corresponding to the YLPG peptide is clearly present. The dotted line indicates the 3 min elution time.

In order to discover the reason for the rapid removal of tetrapeptide from *Xac* cultures, the oligopeptide was incubated with aliquots from the culture supernatant of both *Xac* strains. No tetrapeptide was detected after incubation with culture supernatants of both parent and mutant strains (Figure 3B/D). In addition, EDTA prevented the *in vitro* peptide degradation following incubation with culture supernatants of both *Xac* strains (Figure 3A,C). Such result indicated that the apparent uptake of the tetrapeptide was mainly attributed to the proteolytic attack by secreted proteases produced by the *Xac* 306 strain. Attempts to block protease activity in culture supernatants of actively growing *Xac* strains with EDTA failed, probably due to the large amount of secreted proteases (data not shown).

**Figure 4:**
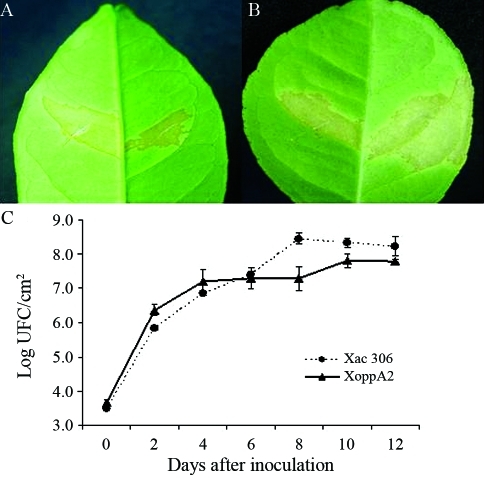
Growth of *Xac* 306 strain and the isogenic *oppA* knockout mutant in susceptible citrus hosts. Leaf lesions caused in (A) sweet oranges(*C. sinensis*) and (B) Rangpur lime (*C. limonia*) after infection with the *Xac* 306 strain (left side) or *XoppA2* strain (right side). Both strains were applied on the abaxial surface of the leaves and photographed 10 days after inoculation. (C) Growth kinetics of the *Xac* 306 and *XoppA2* strains after inoculation in sweet orange leaves*.* Growth of the *Xac* 306 (•) and *XoppA2* strains (▲) was followed during a period of 12 days.

In order to evaluate the putative role of OppA in *Xac* pathogenesis in citrus hosts, we infected two susceptible citrus hosts, *viz*., sweet orange (*C. sinensis*) and Rangpur lime (*C. limonia*), with the *Xac* 306 and *XoppA2* strains, and compared the induction of leaf lesions and multiplication in leaf tissues. Up to 10 days following infection, no difference was detected in the symptoms inflicted by the two *Xac* strains in either citrus host (Figure 4). Furthermore, no significant difference in the growth curves of either of the two strains was detected during their multiplication in sweet orange leaf tissues (Figure 4). These results clearly indicated that deletion of the *oppA* gene did not impair the growth of *Xac* in susceptible citrus hosts.

## Discussion

In the present study we investigated the prevalence of the *oppA* gene, and the corresponding OppA protein, in different *Xanthomonas* species, an important group of phytopathogens inflicting heavy losses in several economically relevant crops. The present results demonstrated that, in contrast to other bacterial groups, such as enterobacterial species and lactic acid bacteria, the *oppA* gene was detected in only two out of the three tested *X. axonopodis* strains. Furthermore, studies screening revealed that *oppA* is also absent in the genomes of the *Xcc*s 8004 and *Xcv* 85-10 strains, as well as two *Xoo* strains with reported genome sequences (Lee *et al.*, 2005; Thieme *et al.*, 2005). We also demonstrated that early-branching *Xanthomonas* species, including the ancestral *X. sacchari* and *X. theicola*, do not carry the *oppA* gene. Similarly, as recently defined by Parkinson and colleagues (2007), other three established *Xanthomonas* phylogenetic groups, represented by *X. bromi*, *X. melonis*, *X. pisi* and *X. codiaei,* also do not carry the *oppA* gene. In spite of our previous observations that the *opp* operon does not present biased codon usage, distinct GC content or the presence of adjacent insertion sequences and transposase-encoding genes (Moutran *et al.*, 2004), the present results indicate that the *opp* genes have been acquired in a recent evolutionary event in the *Xanthomonas* genus and remained restricted to some *X. axonopodis* strains.

Generation of an *oppA*-deficient strain led us to conclude that the Opp system does not play a significant role in the uptake of a tetrapeptide, the most likely substrate of OppA encoded by the *Xac* 306 strain, as previously determined by molecular modeling tools (Moutran *et al.*, 2007). Monitoring of the peptide uptake by mass spectrometry showed that, in contrast to *E. coli*, the tetrapeptide is quickly degraded by secreted proteases produced by both *Xac* 306 and *XoppA2* strains. Hence, it may be deduced that the abundant production of extracellular proteases produced by *Xac*, as well as other *Xanthomonas* species, could constitute an abundant source of amino acids derived from the proteolytic degradation of host proteins. Under such conditions, the function of an oligopeptide uptake system would be dispensable, since free amino acid residues are actively transported by different dedicated uptake systems. This conclusion is supported by the lack of the *oppA* gene in most *Xanthomonas* species and the presence of a stop codon located 129 bp downstream of the first structural codon in the *Xac* 306 *oppD/F* cistron, this encoding the ATPase component required for the generation of energy to the transport process (Silva *et al.*, 2002; Moutran *et al.*, 2004). The finding that eight other *Xanthomonas* species, besides one *Xaa* strain, which do not carry *opp* genes, lends further support to the notion that, in contrast to other bacterial species, *Xanthomonas oppA* genes really represent pseudo genes on the way to disappearing from the genomes of these strains.

In accordance with this idea, no measurable difference in colonization, infection and generation of leaf lesions was observed in two susceptible citrus hosts infected with either the *Xac* 306 or the isogenic *oppA*-deficient strains. The absence of any significant pathogenic impact involving the *Xac* 306 strain in various citric hosts, lends further support to the conception that the Opp system is not functional in this strain and does not contribute to the pathogenesis of this bacterial strain in different citrus hosts. Collectively, the present evidence indicates that the *Xac* OppA, and consequently the Opp system, in contrast to other bacterial species, does not play a relevant physiological role.

## Figures and Tables

**Table 1:** Bacterial strains used in the present study.

Strains	Characteristics	Source or reference
*X. a. citri* 306	Wild type A	Silva *et al.*, 2002
*X. a. citriXoppA2*	*opp*A::*sp/sm*	Oshiro *et al.*, 2006
*X.a*. *aurantifolii* 409	Wild type B	IBSBF 409
*X. a. aurantifolii* 381	Wild type C	IBSBF 381
*X. bromi*	Wild type	LMG 947
*X. c*. *campestris*	Wild type	ATCC 33913
*X. c.vesicatoria*	Wild type	LMG 911
*X. codiaei*	Wild type	LMG 8678
*X. melonis*	Wild type	LMG 8670
*X. pisi*	Wild type	IBSBF 1356
*X. sacchari*	Wild type	LMG 739
*X. theicolai*	Wild type	LMG 8684
*E.coli* K12 SS320	*F*^*-*^*lacI22 lacZ pro-48 met-90 trpA trpR his-85 rpsL azi-9 gyrA* λ^*-*^*P1*^*s*^	Andrews and Short, 1985
*E.coli* K12 SS5013	Δ(*tdk-oppABCDF-tonB-trp*)	Andrews and Short, 1985

ATCC - American Type Culture Collection. IBSBF - Biological Institute Culture Collection of Phytopathogenic Bacteria. BCCM-LMG - Belgian Co-ordinated Collections of Microorganism-Laboratorium voor Microbiologie.
